# Supplementary material for: Expanding the active charge carriers of polymer electrolytes in lithium-based batteries using an anion-hosting cathode
Source: Nat Commun. 2022 Jun 9;13:3209. doi: 10.1038/s41467-022-30788-5 (PMC9184592; doi:10.1038/s41467-022-30788-5)
Supplement: Supplementary file 1 — Supplementary Information [file 41467_2022_30788_MOESM1_ESM.pdf]

Supplementary Information

Expanding the active charge carriers of polymer electrolytes in lithium-based batteries using an anion-hosting cathode

Zongjie Sun[1]†, Kai Xi[1]†, Jing Chen[1], Amor Abdelkader[2], Meng-Yang Li[1], Yuanyuan Qin[1], Yue Lin[3], Qiu Jiang[4], Ya-Qiong Su[1], R. Vasant Kumar[5], Shujiang Ding[1]*

[1]School of Chemistry, Engineering Research Center of Energy Storage Materials and Chemistry for Universities of Shaanxi Province, State Key Laboratory for Mechanical Behavior of Materials, Xi'an Jiaotong University, Xi'an 710049, China.

[2]Faculty of Science and Technology, Bournemouth University, Talbot Campus, Fern Barrow, Poole, BH12 5BB, United Kingdom

[3]Cavendish Laboratory, University of Cambridge, Cambridge CB3 0HE, United Kingdom

[4]School of Materials and Energy, University of Electronic Science and Technology of China, Chengdu 611731, China

[5]Department of Materials Science and Metallurgy, University of Cambridge, Cambridge CB3 0FS, United Kingdom.

* Corresponding author. Email: *dingsj@mail.xjtu.edu.cn*

This file includes:

Supplementary Note 1

Supplementary Figure 1 to 33

Supplementary Table 1 to 6

Supplementary Note 1: Shape of cyclic voltammograms with PVF cathode

All CVs in main text Figure. 4a correspond to reversible redox. The repeatability suggests that impurities are unlikely to be the source of the non-ideal behavior. The CV of an ideal surface binding reaction contains a single peak. However, some anion (typically, $TFSI^-$) pairs exhibit deviations from ideal behavior, with asymmetry/multimodality and peak broadening. There are theoretically potential origins for the anion-related properties of peak shape, including:

(1) Buried Fc and plane of electron transfer (PET). Active unit (ferrocene) packing in PVF buries part of the Fc. As described in the previous experiments and model about close-packed Fc self-assembled monolayers (SAMs)[1,2], the positional difference between the active unit and the closest ion/ion cluster creates multiple electron transfer planes (PETs) that induce splitting and broadening of the CV peak. We noticed that making the units farther apart (VFS cathode with $TFSI^-$ anion, shown in **Supplementary Figure 11**) obtained a more ideal CV.

(2) Intermolecular interactions experienced by the Fc and anions. For Fc surface-bound redox activity, CVs can be fitted to models based on Langmuir or Frumkin isotherms to gain insight into the nature of intermolecular interactions[3,4], including oxidized and reduced forms of Fc with anions. In addition, differences in anion size make internal transport potentially sterically hindered, leading to discriminatory CV results[5].

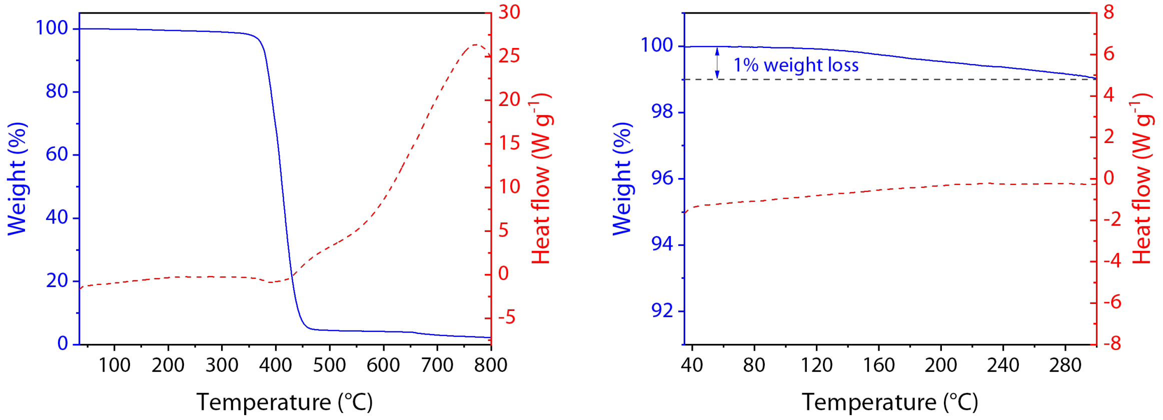

Supplementary Figure 1. TGA and DSC curves of the prepared PVF. The polymer has almost no mass loss before pyrolysis at 400 °C.

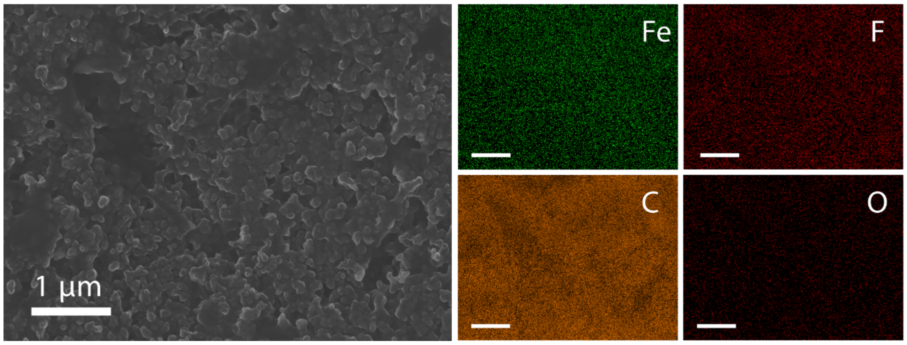

Supplementary Figure 2. The SEM and EDX elemental mapping of cathode (PVF: Super P: PVDF = 4: 5: 1, mass ratio), the scale bar in all EDX results is 1 µm, same as SEM.

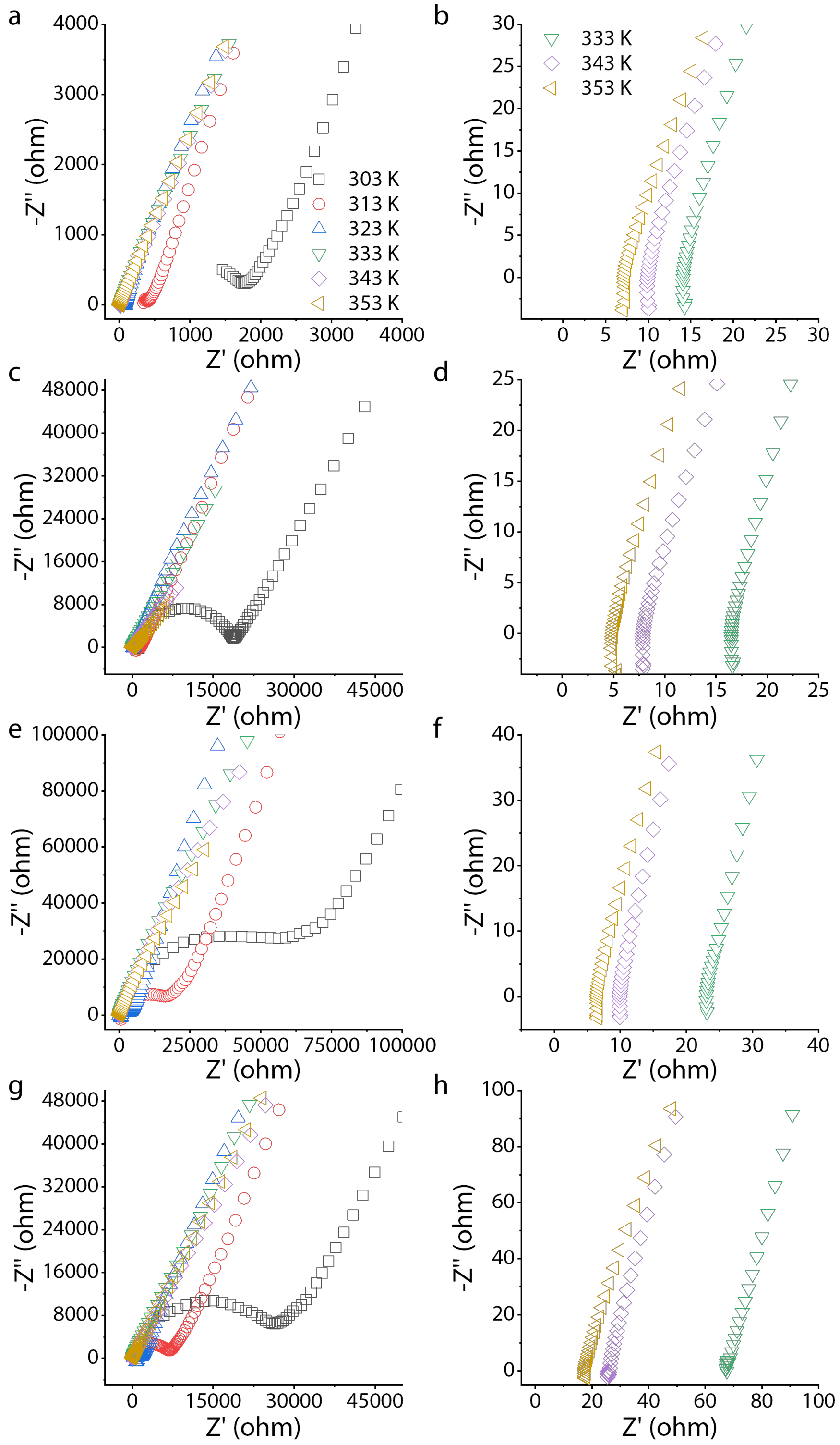

Supplementary Figure 3. Nyquist plots of SPEs at different temperatures (O: Li = 20: 1), test using stainless steel|SPE|stainless steel structure. (**a**, **b**) LiTFSI-PEO (**c**, **d**) LiBOB-PEO (**e**, **f**) LiFSI-PEO (**g**, **h**) $LiClO_4$-PEO.

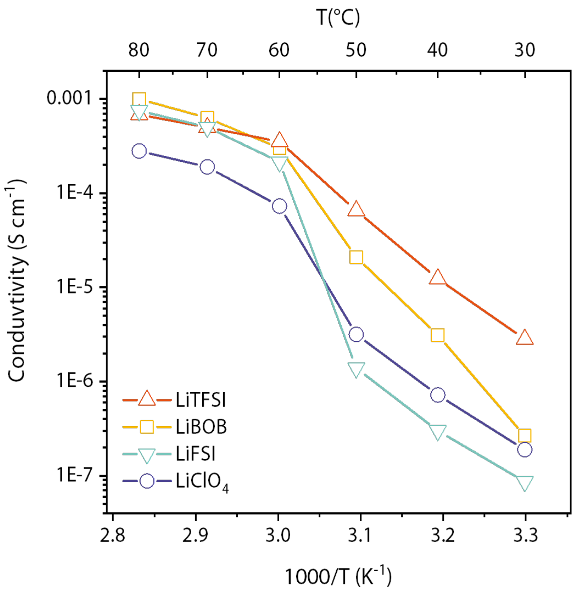

Supplementary Figure 4. Ionic conductivity results of SPEs. The ion conductivity changes of different salts with temperature, calculated from Supplementary Figure 3.

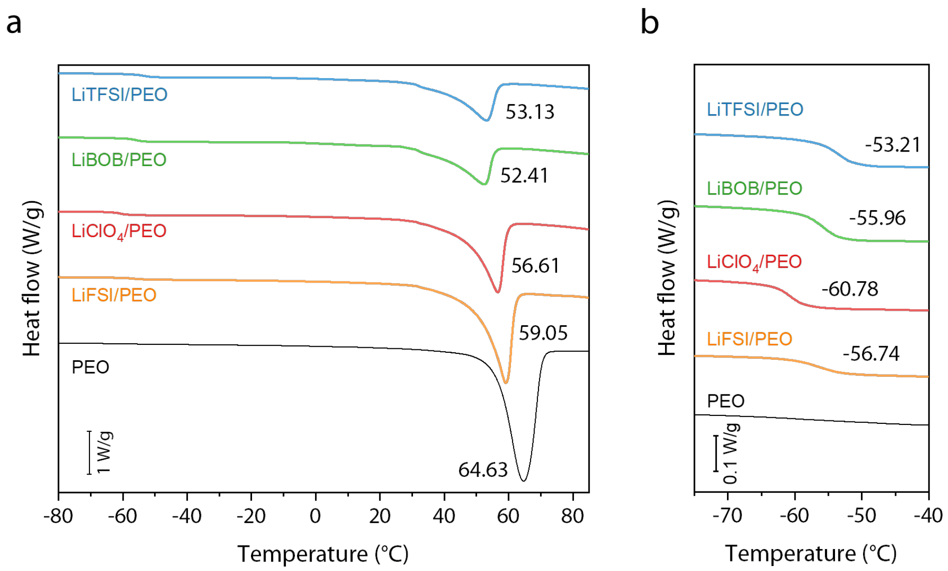

Supplementary Figure 5. The DSC curves of SPEs. Using LiTFSI and LiBOB as salts, the melting enthalpy of SPEs decrease significantly. However, $LiClO_4$ and LiFSI have a weak effect on reducing polymer crystallinity.

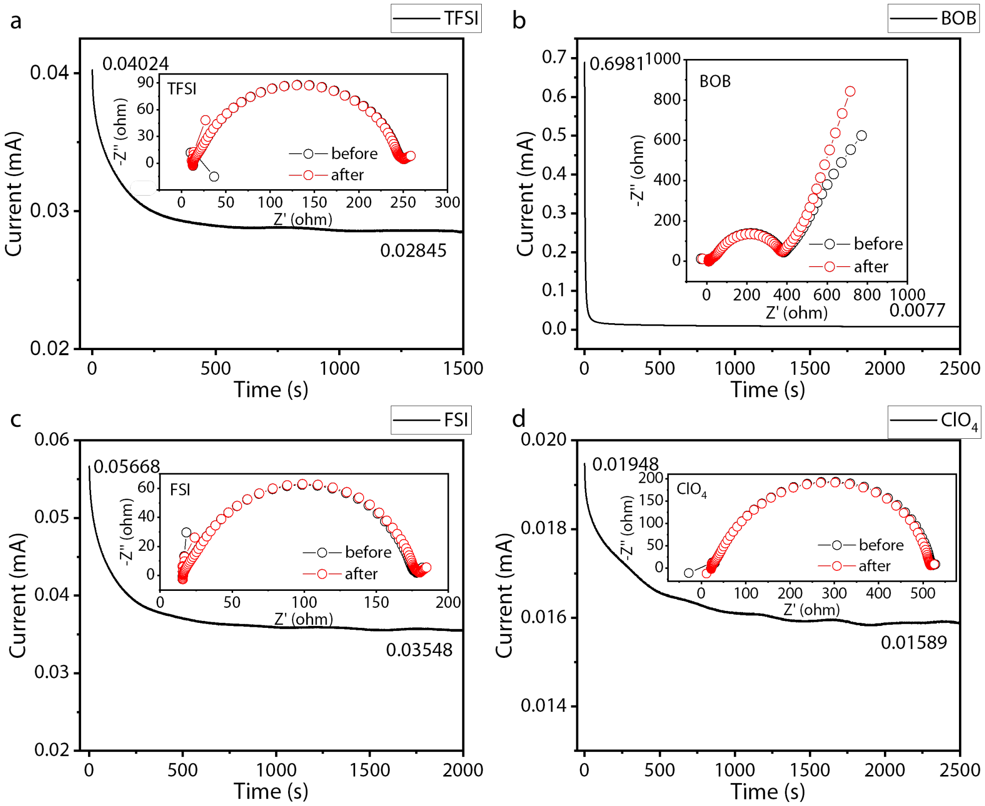

Supplementary Figure 6. Determination of lithium-ion migration number. Chronoamperometry profiles and AC impedance spectra before/after polarization for symmetric Li||Li cells at 60 °C, with (**a**) PEO-LiTFSI (**b**) PEO-LiBOB (**c**) PEO-LiFSI and (**d**) PEO-$LiClO_4$ as electrolyte.

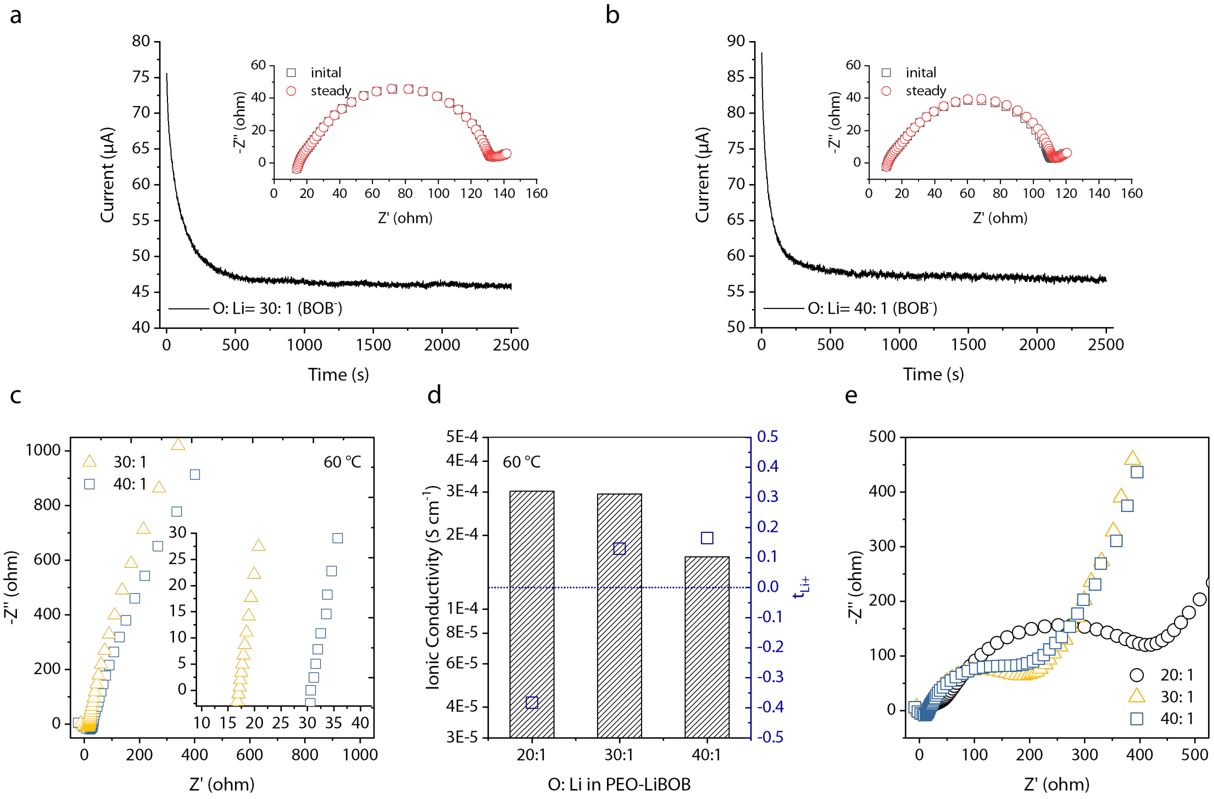

Supplementary Figure 7. Salt concentration impact in PEO-LiBOB. Chronoamperometry profiles and electrochemical impedance spectra for symmetric Li|PEO-LiBOB|Li cells with O: Li at (**a**) 30: 1 and (**b**) 40: 1. (**c**) AC impendence, (**d**) ionic conductivity and t_{Li+} of SPEs. (**e**) Nyquist plot of Li|PEO-LiBOB|PVF cells with different O: Li. All the electrochemical tests were performed at 60°C.

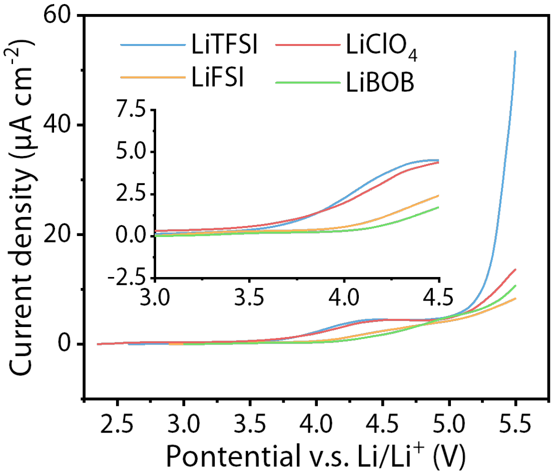

Supplementary Figure 8. Linear sweep voltammetry of SPEs. Tested using CR2025 coin-cell configuration with Li|SPE|stainless steel at 60 °C, 0.2 mV s^{-1}.

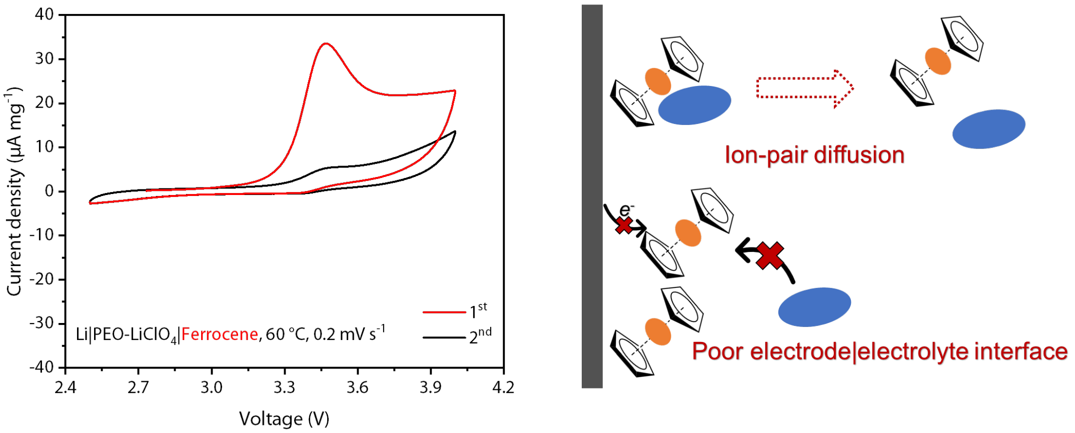

Supplementary Figure 9. Redox properties of ferrocene. The CV results of Li||ferrocene cell with PEO-$LiClO_4$ electrolyte at 60 °C, 0.2 mV s^{-1}. The prominent oxidation peak observed in the first cycle appears as a hump in the second cycle, indicating the poor reversibility of the ferrocene redox.

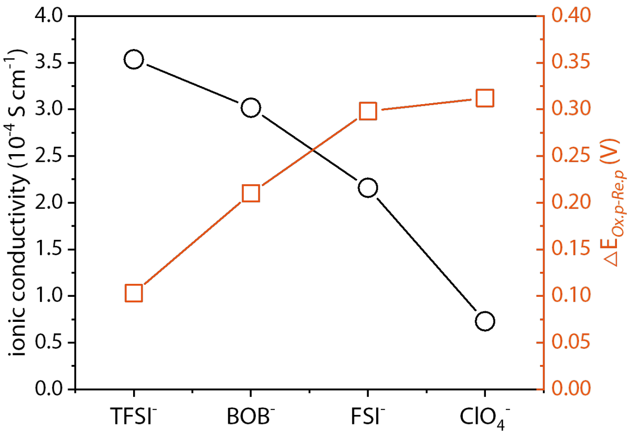

Supplementary Figure 10. The ionic conductivity and redox peaks difference of SPEs (ΔE, collected from CV results) with anion species at 60 °C

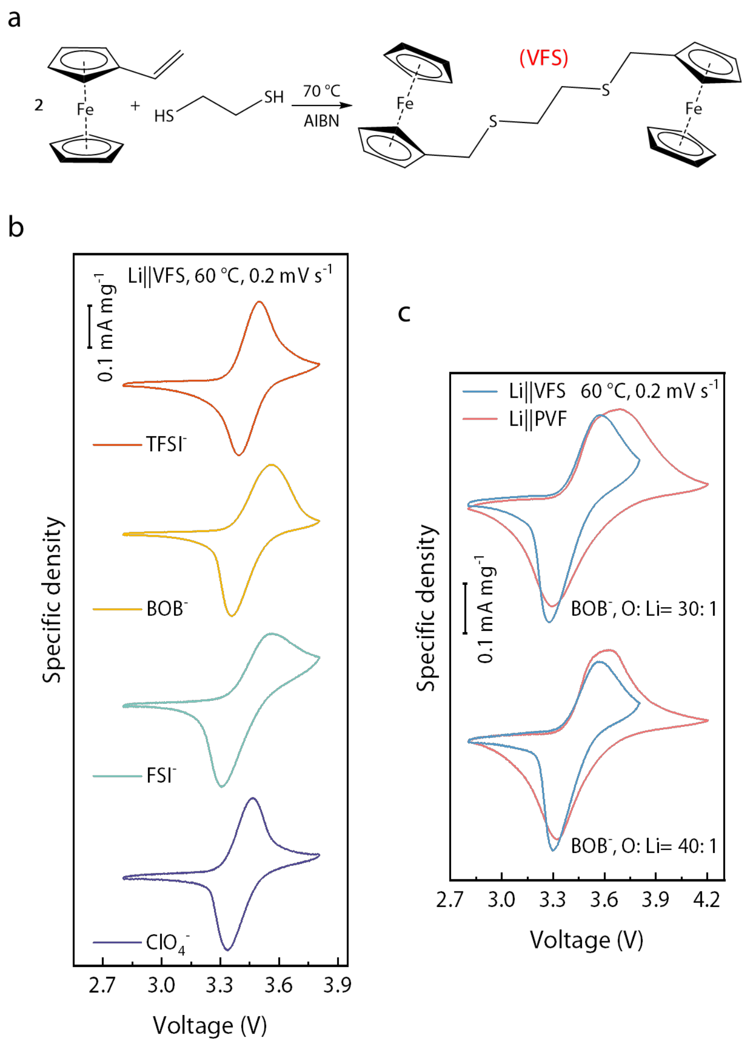

Supplementary Figure 11. Synthesis and electrochemical tests of liner structure ferrocene cathode. (**a**) synthesis of liner structure ferrocene (VFS) by click-chemistry. Specifically, 1 g of vinyl ferrocene a 1.5-fold molar amount of ethanedithiol (99%, Aladdin) were put into 5 ml of dry toluene. The reaction was stirred at 70 °C for 24 hours, and the resulting solution was precipitated with methanol. The mass ratio of active materials (VFS): Super P: PVDF = 4: 5: 1 in electrode. (**b**), (**c**) show the CV results with Li||VFS cells at 60 °C. Various anion species and the ratio of O: Li in PEO-LiBOB were investigated (scan rate, 0.2 mV s^{-1}). During the tests, the VFS electrode showed extra oxide current beyond 3.8 V due to the instability of -C-S-C- bond. Thus, the CV measurements were performed at 2.6~3.8 V for Li||VFS cells.

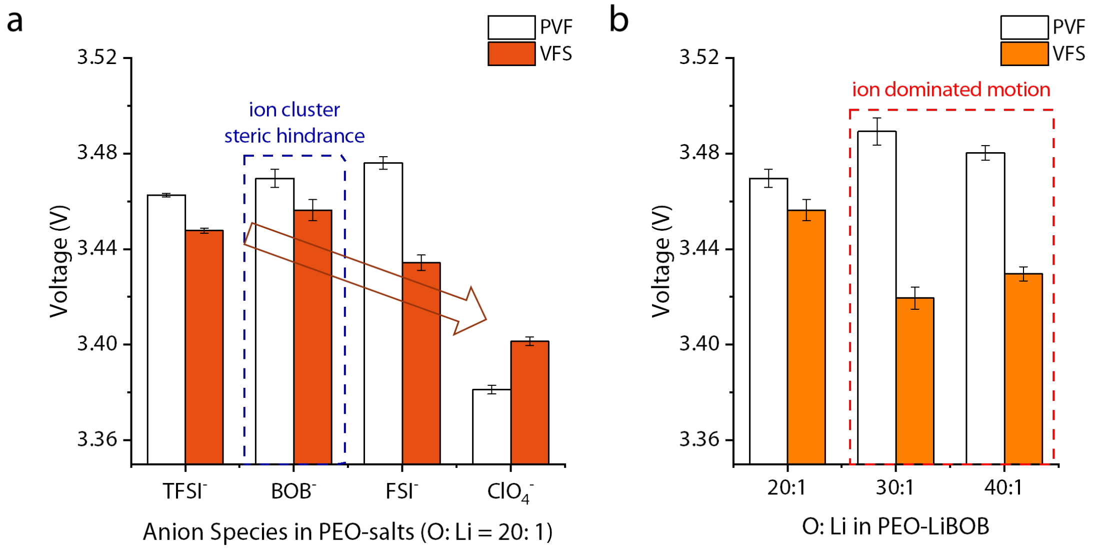

Supplementary Figure 12. E_0 comparison with PVF and VFS as the cathode. The impact of (**a**) anion species and (**b**) the ratio of O: Li in PEO-LiBOB on E_0. Error bars represent the standard deviation calculated by the data sets. During the CV test of Li||VFS cells, except for PEO-LiBOB, the electrode potential (E_0) decreases at the order of binding energy, matching the computation results. The E_0 drop of BOB^--based reaction has a specific deviation from binding energy. This result can be attributed to the steric effect of anion-dominated ion clusters. For the O: Li ratios at 30: 1 and 40: 1 in BOB^--based reaction, the E_0 decrease of the VFS to PVF is significant, proving the steric effect from anion side. The above results show that the steric hindrance of large anions ($TFSI^-$, BOB^-, FSI^-) with PVF and the ion clusters (BOB^- at 20: 1) dominated hindrance both significantly impact the reaction potential.

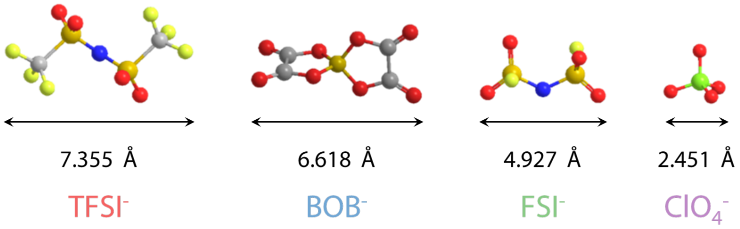

Supplementary Figure 13. The structures and sizes of anions used in this work. Collected from the two farthest atoms in the molecule.

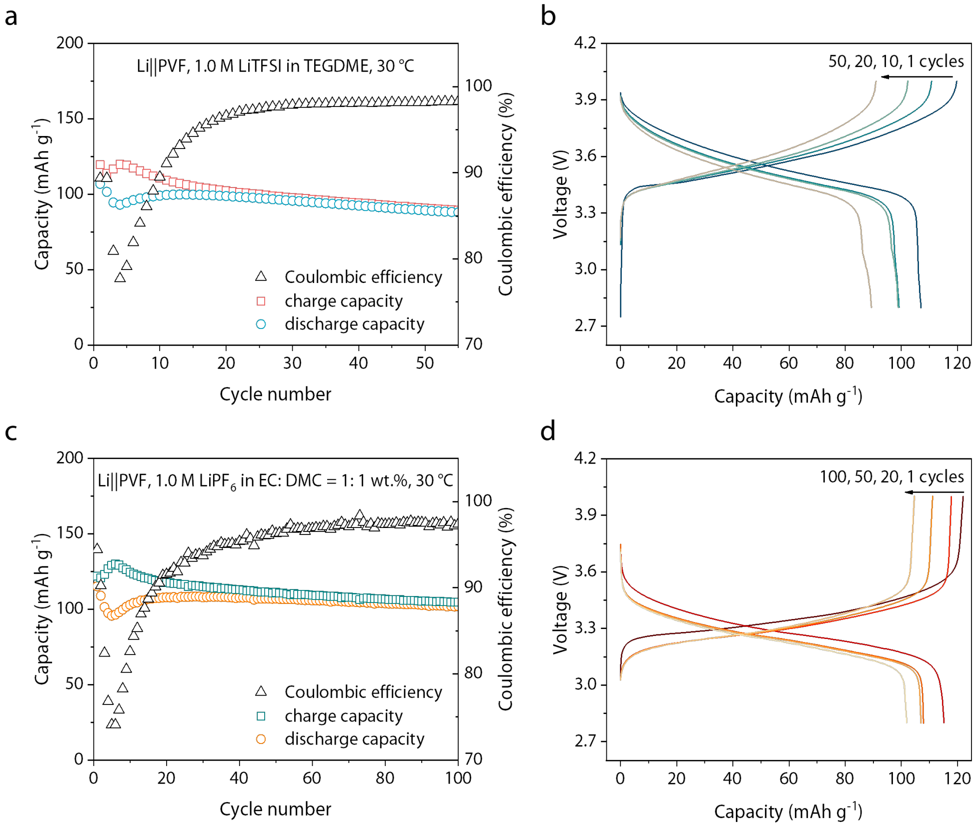

Supplementary Figure 14. The cycles of Li||PVF in liquid electrolyte, specific current applied of 62 mA g^{-1} at 30 °C. The capacity and potential profiles of cells at (**a**, **b**) 1.0 M LiTFSI in TEGDME and (**c**, **d**) 1.0 M $LiPF_6$ in EC: DMC = 1: 1 wt.%.

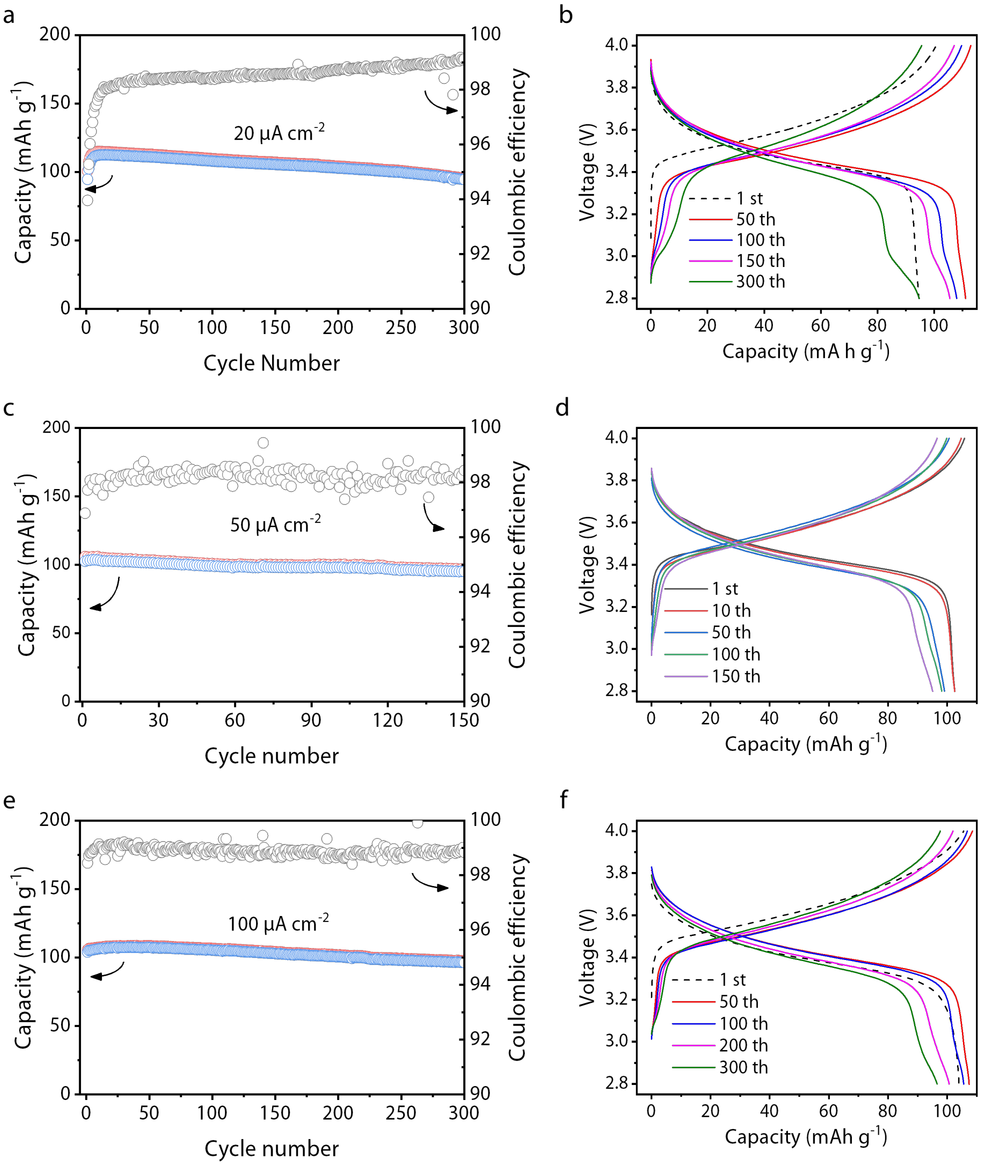

Supplementary Figure 15. Cycle performance of Li|PEO-LiBOB|PVF cells at 60 °C. The capacity, coulombic efficiency and potential profiles of cells at (**a**, **b**) 20, (**c**, **d**) 50 and (**e**, **f**) 100 μA cm^{-2}.

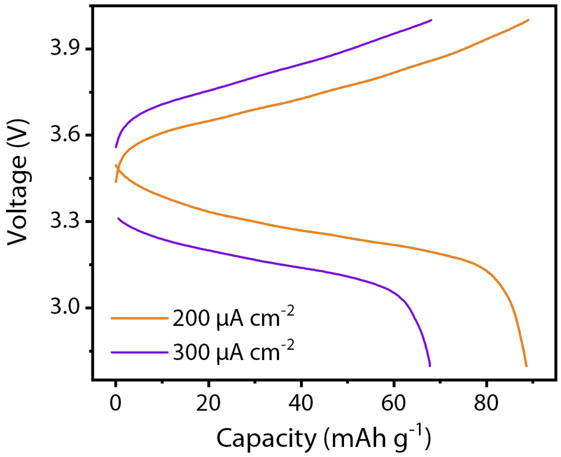

Supplementary Figure 16. Potential profiles of Li|PEO-LiBOB|PVF full cells at 200, 300 μA cm^{-2} and 60 °C.

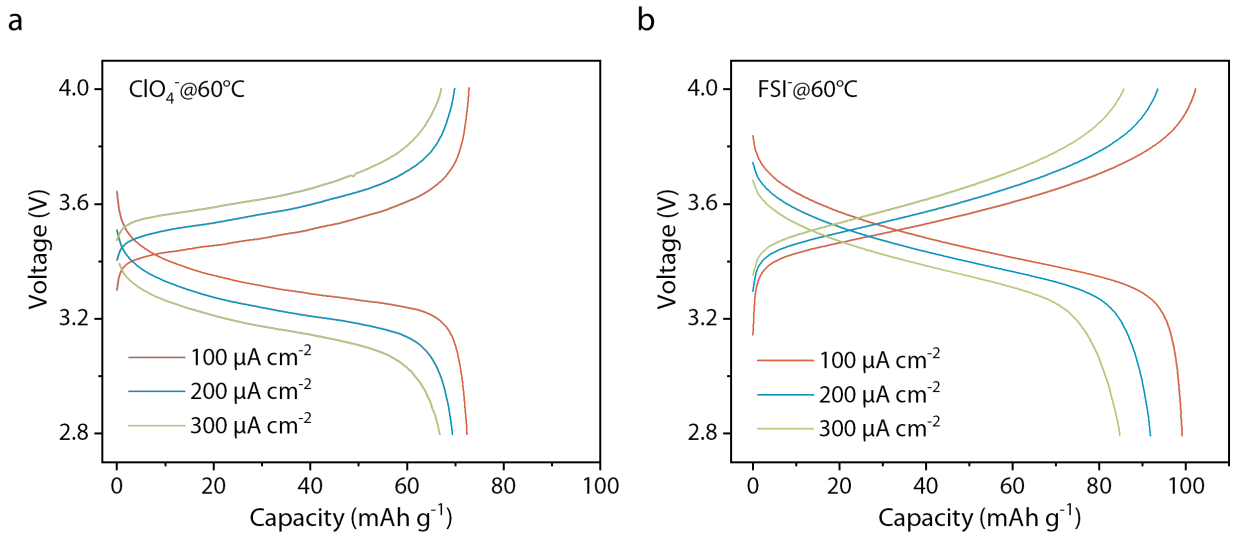

Supplementary Figure 17. Potential profiles of (**a**) Li|PEO-LiClO$_4$|PVF and (**b**) Li|PEO-LiFSI|PVF full cells at 60 °C.

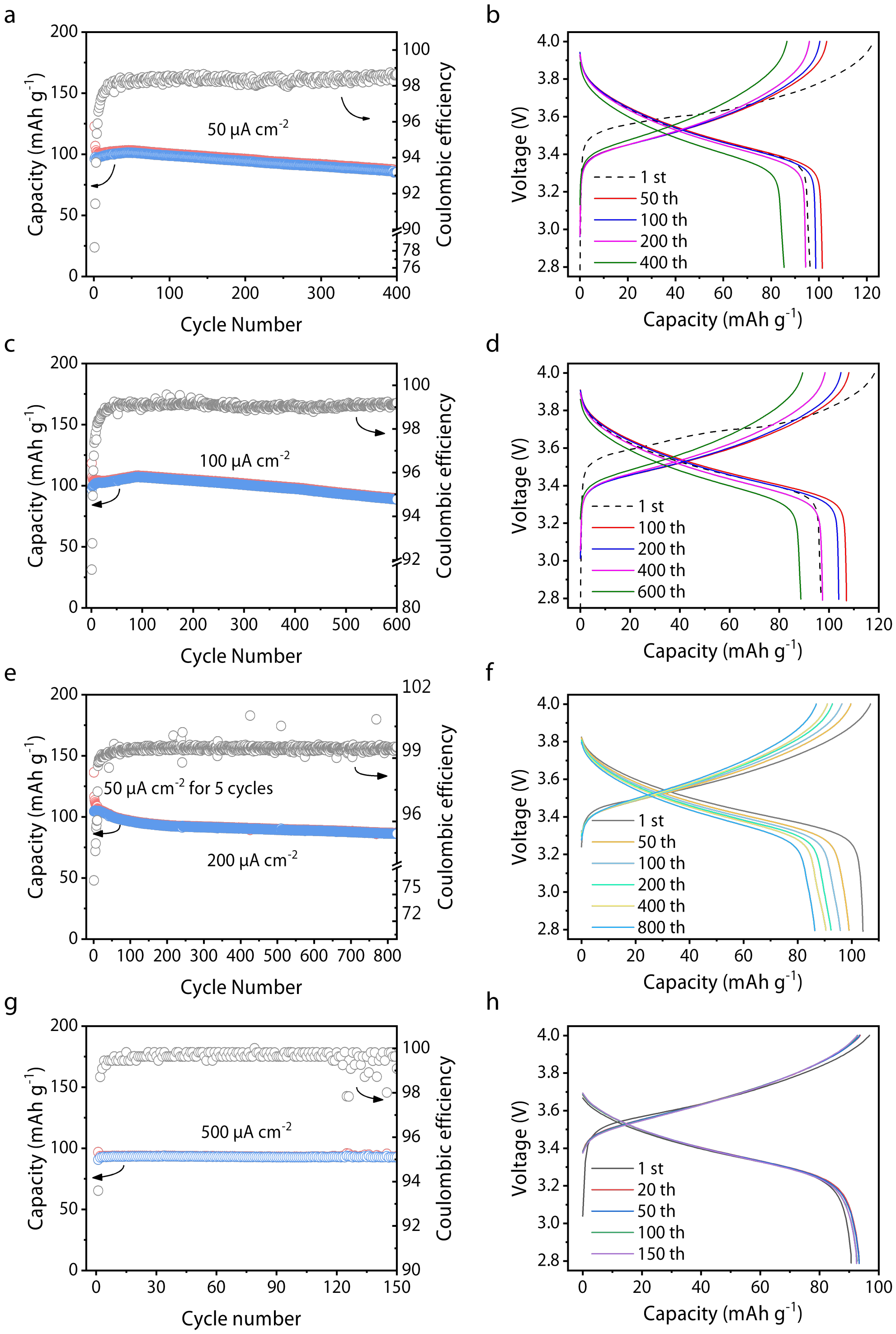

Supplementary Figure 18. Cycle performance of Li|PEO-LiTFSI|PVF cells at 60 °C. The capacity, coulombic efficiency and potential profiles of cells at (**a**, **b**) 50, (**c**, **d**) 100, (**e**, **f**) 200 and (**g**, **h**) 500 μA cm^{-2}.

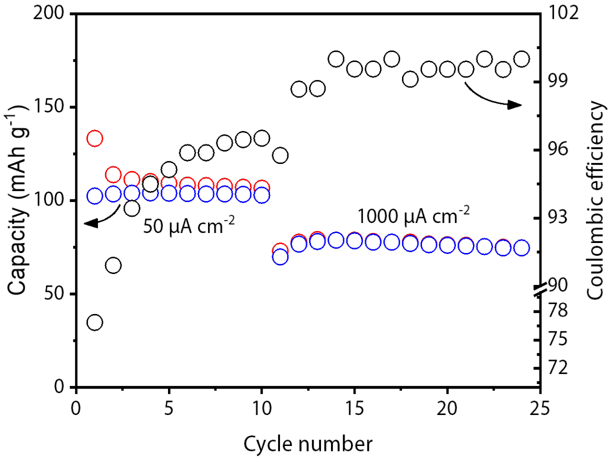

Supplementary Figure 19. The capacity and coulombic efficiency of Li|PEO-LiTFSI|PVF at 1000 μA cm^{-2}, 60 °C.

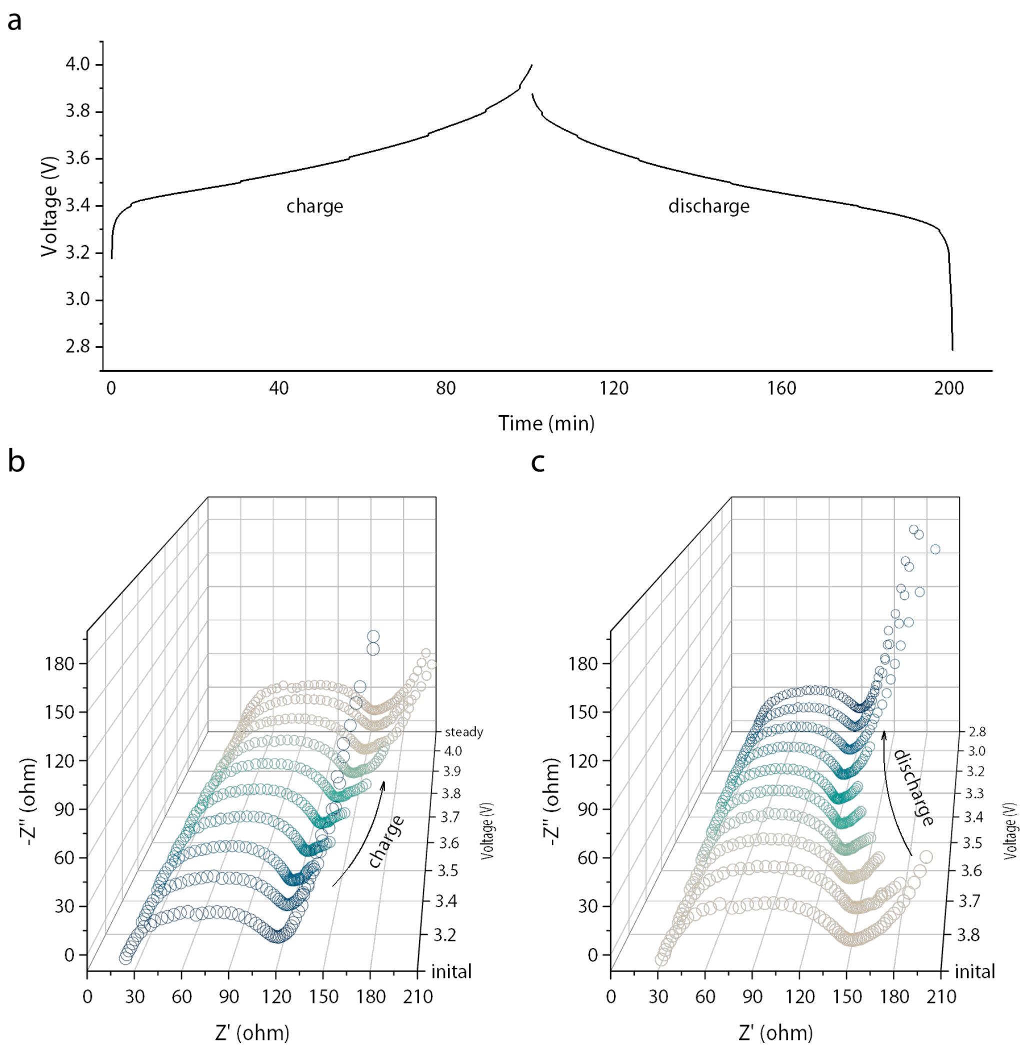

Supplementary Figure 20. (**a**) Potential profile and (**b**, **c**) EIS of Li|PEO-LiTFSI|PVF at 60 °C, 100 μA cm^{-2}.

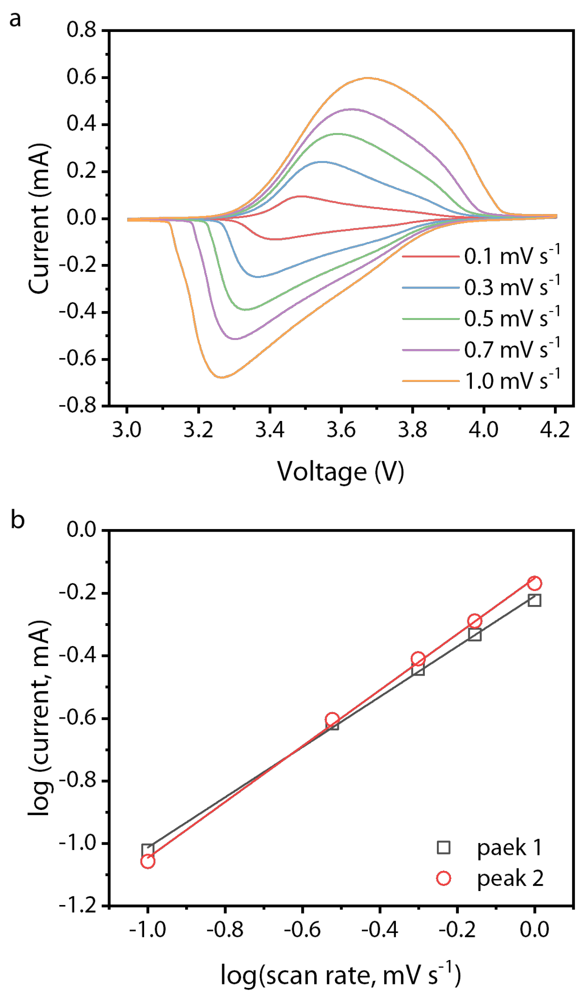

Supplementary Figure 21. Charge storage behaviour in Li|PEO-LiTFSI|PVF cell, performed at 60 °C. Generally, the electrochemical kinetics processes obey the following equations[6].

$$i = av^b \quad (1)$$

$$\log(i) = b\log(v) + \log(a) \quad (2)$$

where ***i*** and ***v*** represent the peak current and corresponding scan rate, respectively, **a** and **b** denote adjustable parameters. The ***b*** value is calculated from the slope of the **log(*i*)** versus **log(*v*)** plot. Generally, the electrochemical process is ionic diffusion dominated when ***b*** approaches 0.5. When ***b*** is close to 1.0, the process is surface-controlled. According to the calculation, the ***b*** values of peak 1 and peak 2 are **0.80** and **0.89**.

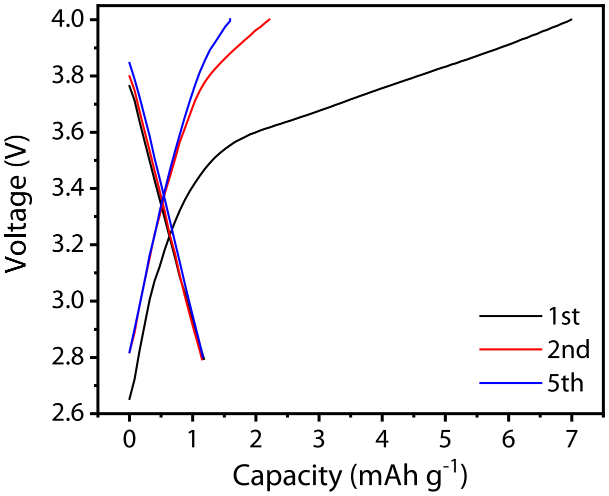

Supplementary Figure 22. Potential profiles of Li|PEO-LiTFSI|Super P cell at 50 μA cm^{-2}, 60 °C. Super P: PVDF = 85: 15 wt.% in electrode. The result confirmed that the capacity contribution of the conductive agents is negligible.

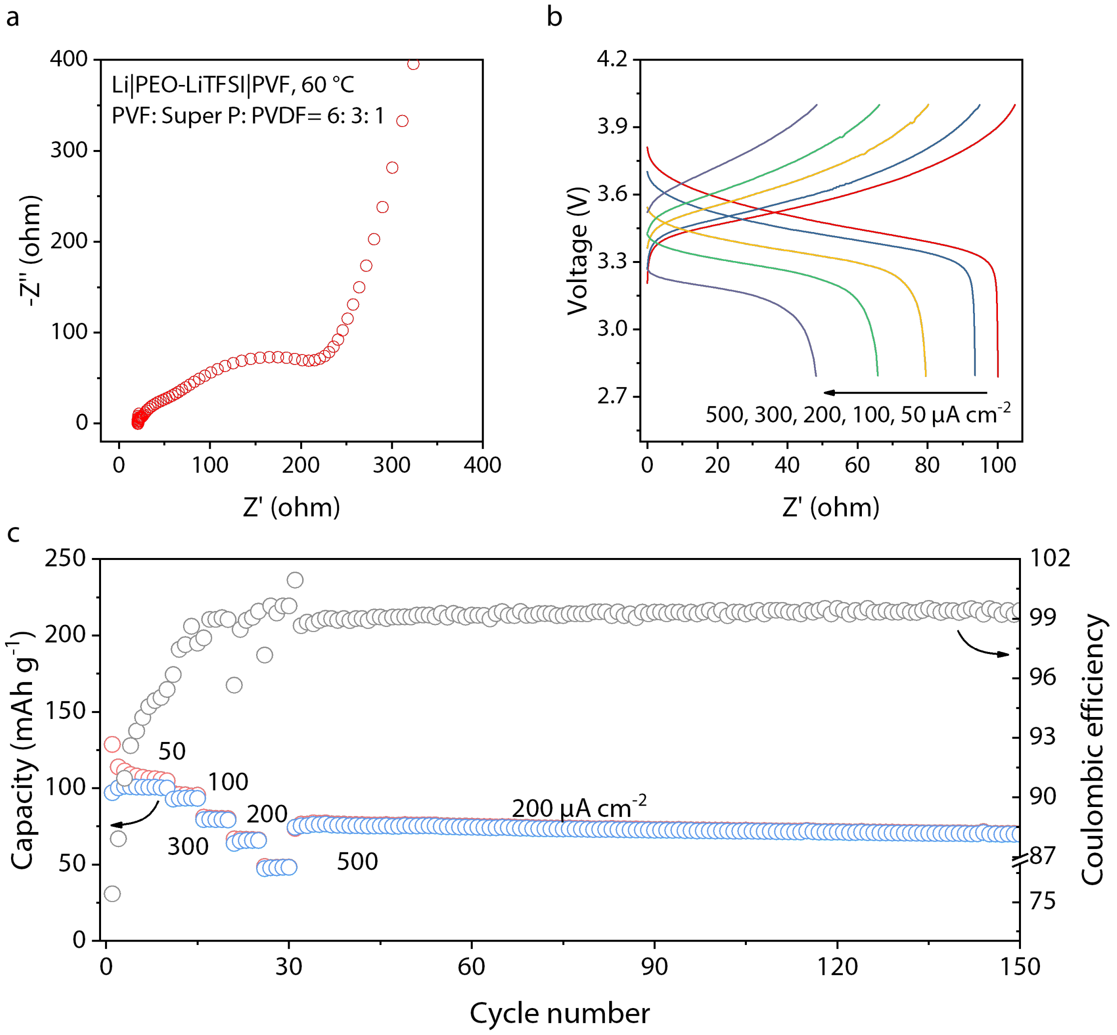

Supplementary Figure 23. The effect of conductive agent content, tested by Li|PEO-LiTFSI|PVF cells at 60 °C. The mass ratio of PVF: conductive agent: binder = 6: 3: 1 (**a**) Nyquist plots of initial cells, (**b**) potential profiles and (**c**) cycle performance of the cell at different current densities.

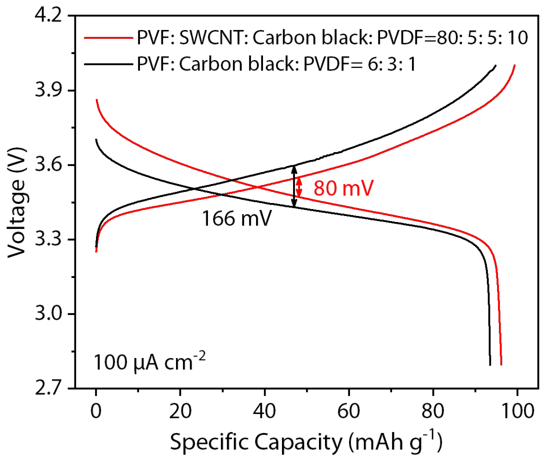

Supplementary Figure 24. Cell performance of SWCNT as the conductive agent, tested by Li|PEO-LiTFSI|PVF cells at 100 μA cm^{-2}, 60 °C. It can be clearly observed that at high active material content (80 wt.%), the electrode with 5 wt.% SWCNT has a lower polarization, drops from 166 mV to 80 mV.

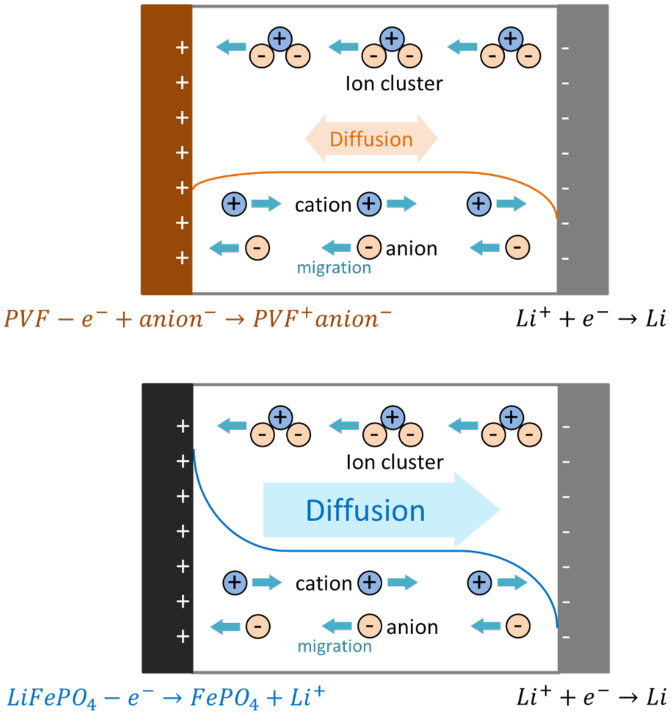

Supplementary Figure 25. Schematic of the migration and diffusion of ions/ion clusters in SPEs with $LiFePO_4$ and PVF as cathode. Take the charging process as an example. The bottom part indicates the reaction of the electrode, and the solid line inside represents the concentration of the substance (ions and ion clusters) in the SPEs.

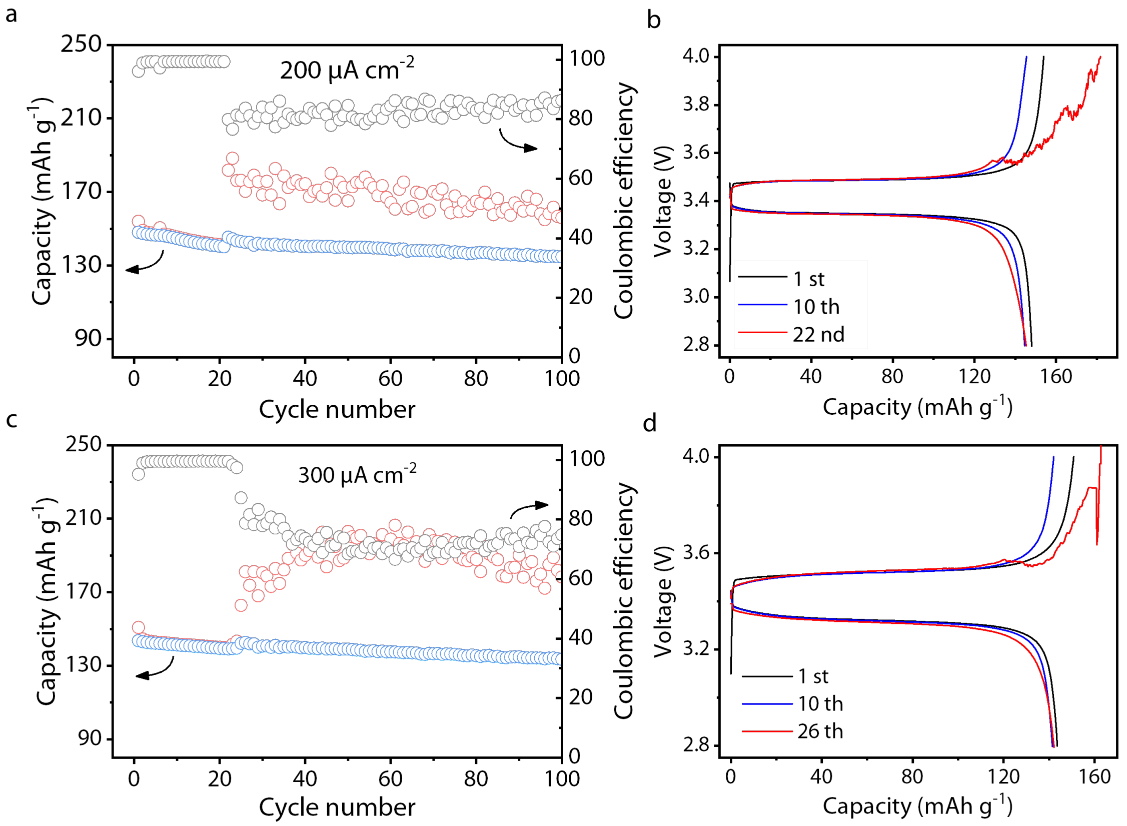

Supplementary Figure 26. Performance of Li|PEO-LiTFSI|$LiFePO_4$ cells at 200 and 300 μA cm^{-2}, 60 °C. (**a**, **c**) capacity, coulombic efficiency and (**b**, **d**) potential profiles of cells.

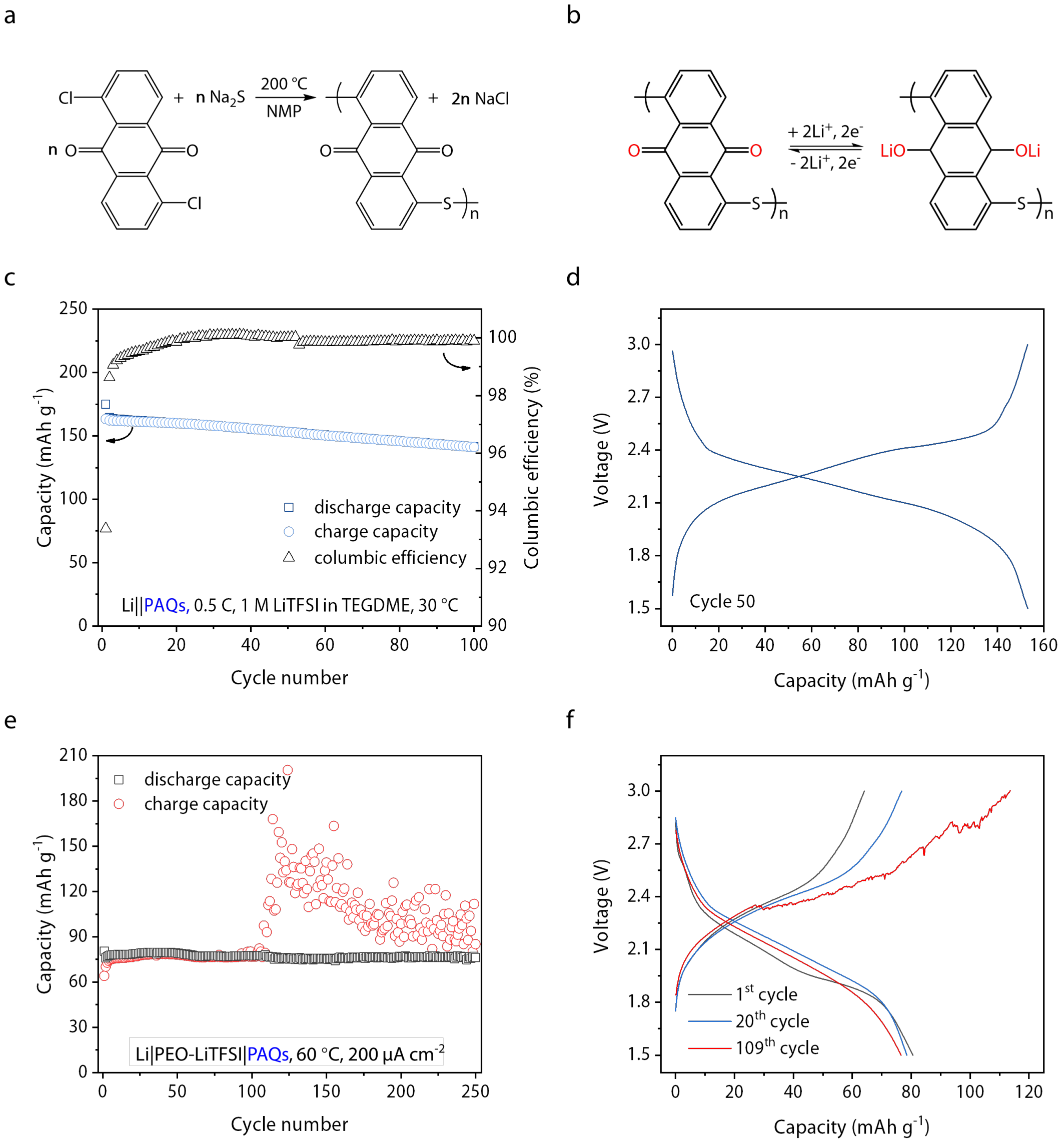

Supplementary Figure 27. Performance of Li||PAQs cell. (**a**) Synthesis of poly(anthraquinonyl sulfide) (PAQs), the methods refer to the previously reported literature[7]. The mass ratio of PAQs: Super P: PVDF = 4: 5: 1 in electrode. (**b**) Redox of PAQs in Li||PAQs cells. The capacity and potential profiles of Li||PAQs cells with (**a**, **b**) 1.0 M LiTFSI in TEGDME, 30 °C and (**c**, **d**) PEO-LiTFSI, 60 °C as electrolyte.

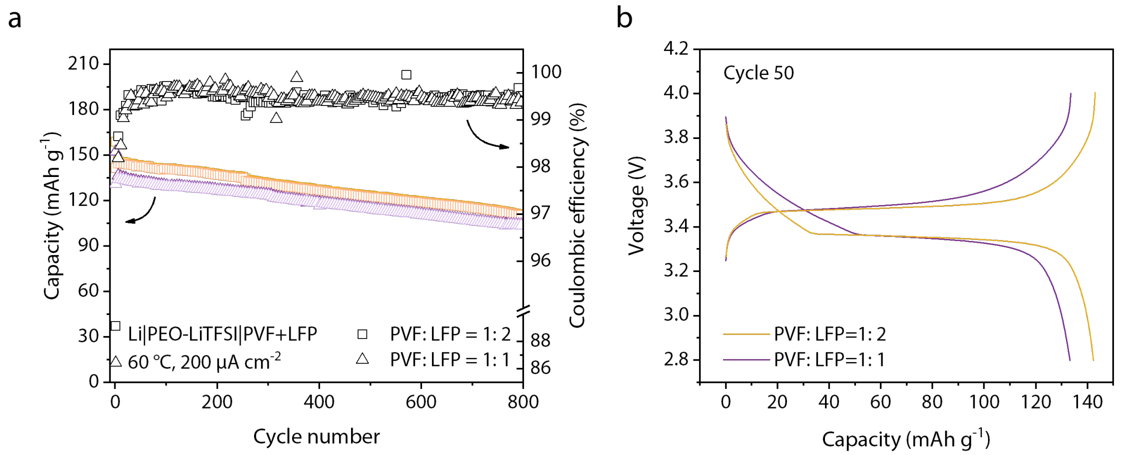

Supplementary Figure 28. Cycles of composite cathodes at 200 µA cm-2, 60 °C. (**a**) Capacity and (**c**) potential profiles of Li|PEO-LiTFSI|PVF+LFP cells (mass ratio of active materials: Super P: PVDF = 6: 3: 1). The mass loading of active materials = 1.5 mg cm^{-2}.

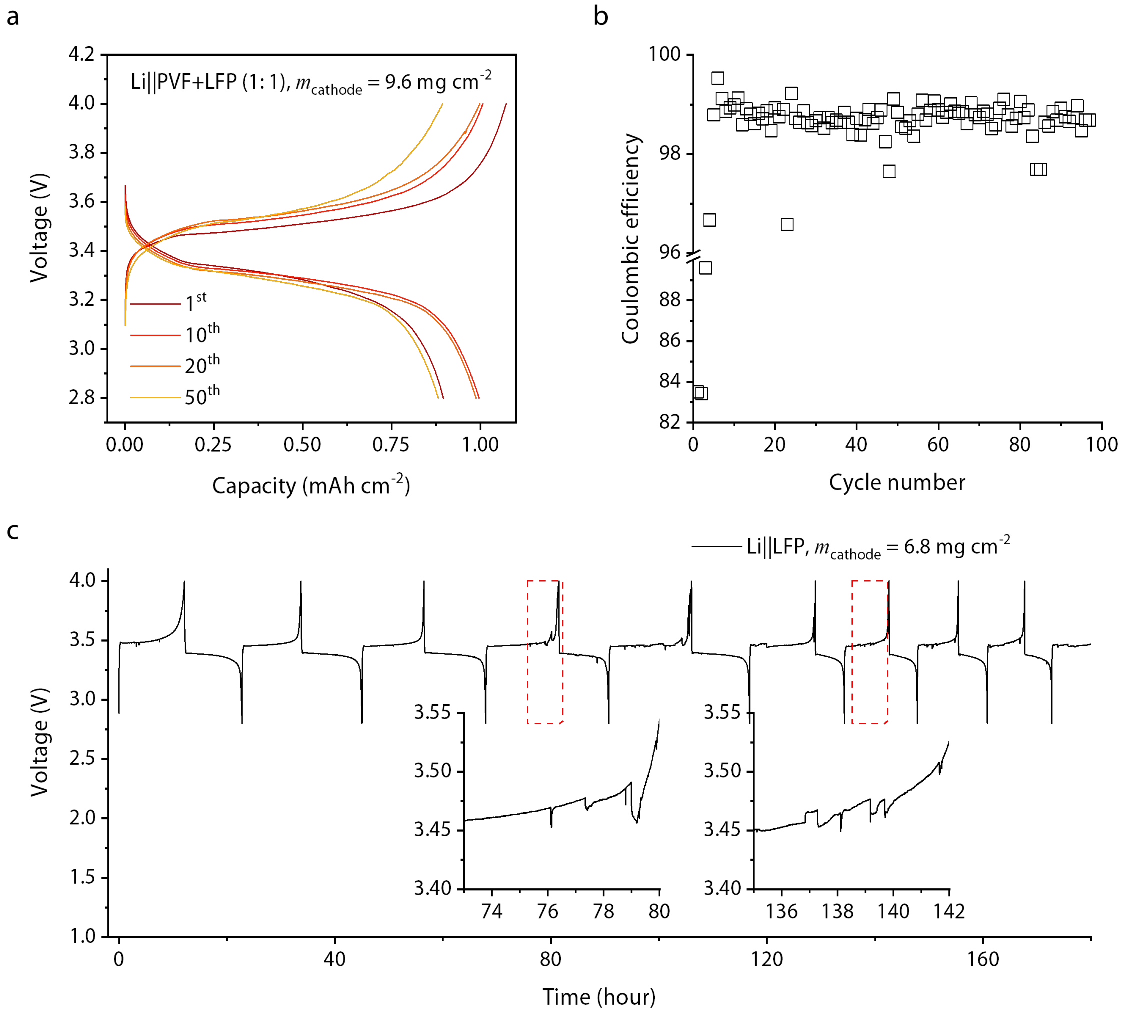

Supplementary Figure 29. Electrochemical energy storage testing of PVF+LFP and LFP cathode with high mass loading. The initial area capacity is limited to around 1 mAh cm^{-2}, the mass loading in figure refer to the active materials. (**a**) and (**b**) show the potential profiles and coulombic efficiency of Li||PVF+LFP cell. (**c**) provide the potential profiles of Li||LFP cell. Benefiting from anion-extended carriers, the mixed cathode provides stable cycles, even if PVF represents 50 wt.% of the active material. In contrast, LFP cathode suffer an irregular voltage fluctuation at 4th cycle, leading to a continuous drop in coulombic efficiency. (Li||LFP cell performed at 100 μA cm^{-2} for first 5 cycles and 200 μA cm^{-2} for the rest, and Li||PVF cell was conducted at 200 μA cm^{-2}, both cells cycled with PEO-LiTFSI electrolyte, 60 °C. The mass ratio of both cathodes was active material: Super P: PEO = 6: 3: 1, PVF: LFP = 1: 1 in the mixed cathode.)

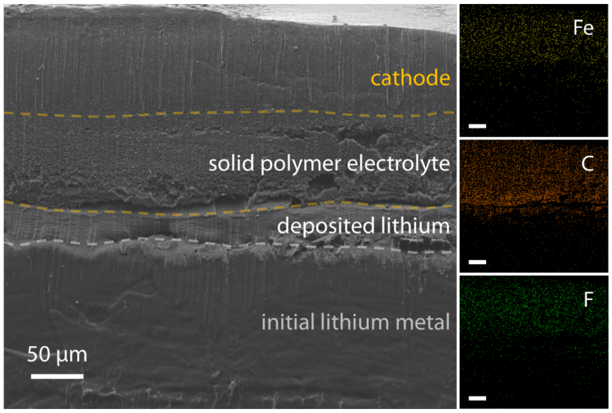

Supplementary Figure 30. Ex situ postmortem SEM and EDX cross-section measurements of the Li|PEO-LiTFSI|PVF+LFP cell after 50 cycles at 200 µA cm^{-2}, 60 °C, area specific capacity around 1 mAh cm^{-2}. The scale bar in all EDX results is 50 µm, same as SEM.

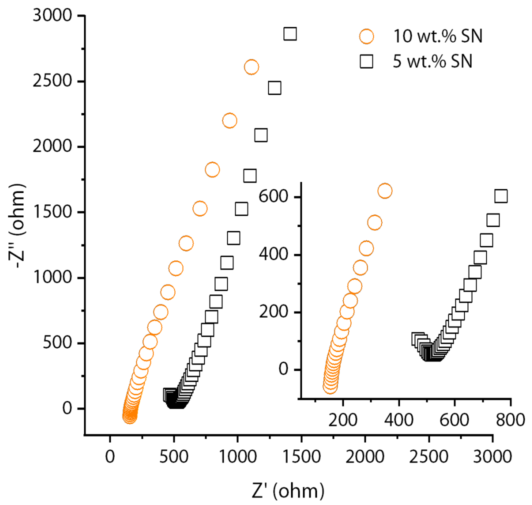

Supplementary Figure 31. Electrochemical impedance spectroscopy of stainless steel|PEO-LiTFSI|stainless steel with 5, 10 wt.% SN content in SPE at 30 °C.

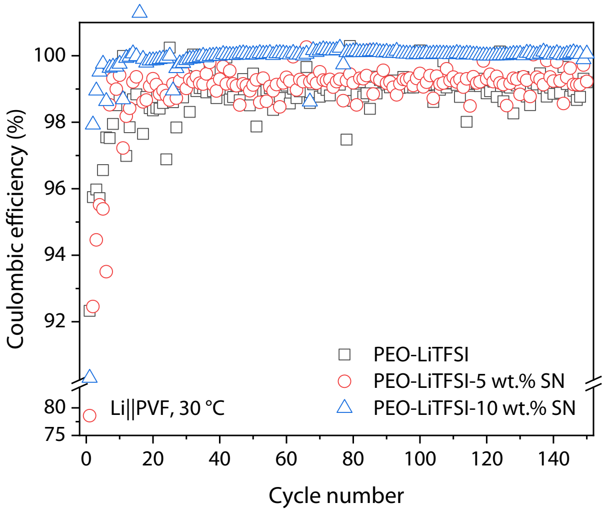

Supplementary Figure 32. Coulombic efficiency of Li|SPEs|PVF cells at 30 °C for rate test in Figure 6a, without/with SN content in SPEs.

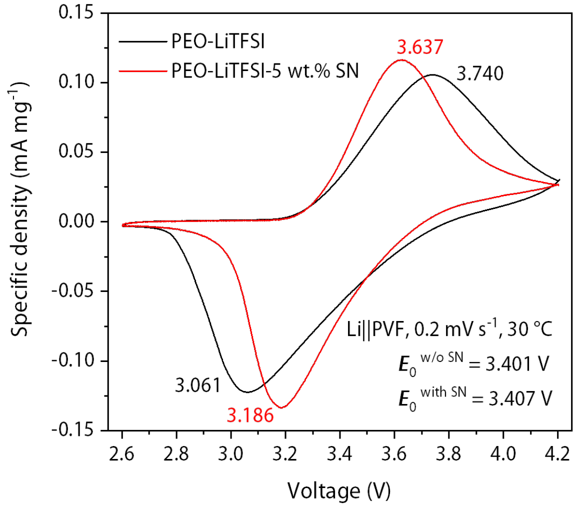

Supplementary Figure 33. CV results of Li||PVF cells without/with 5 wt.% SN in SPEs at 0.2 mV s^{-1}, 30 °C.

Supplementary Table 1. Characterization for polymerization

monomer	initiator	monomer /initiator (mole ratio)	time (h)	conversion (%)	M_n (g mol^{-1}) [a)]	M_w (g mol^{-1}) [a)]
vinyl ferrocene	2,2′-azobis (2-methylpropionitrile)	100	48	38	4815	8222

[a)] The M_n (number-average molecular weight) and M_w (weight-average molecular weight) were obtained by gel permeation chromatograph in tetrahydrofuran, M_n / M_w = 1.708.

Supplementary Table 2. Determination of lithium ion transference number by chronoamperometry

	$\boldsymbol{I_0}$ (mA)	$\boldsymbol{I_{SS}}$ (mA)	$\boldsymbol{R_0}$ (Ω)	$\boldsymbol{R_{SS}}$ (Ω)	$\boldsymbol{t_{Li^+}}$
PEO-LiTFSI	0.04024	0.02845	238.07	237.70	0.092
PEO-LiBOB	0.6981	0.0077	372.03	365.69	-0.383
PEO-LiFSI	0.05668	0.03548	162.53	164.45	0.118
PEO-$LiClO_4$	0.01948	0.01589	499.22	495.23	0.105

Supplementary Table 3. Equivalent circuit and fitting results of Li|SPE|PVF cells

SPE of Li‖PVF cells	equivalent circuit[b)]	R_0 (Ω)	R_{ct} (Ω), mid-frequency	$R_{ct'}$ (Ω), high-frequency
PEO-LiTFSI		15.60	99.32	/
PEO-LiFSI	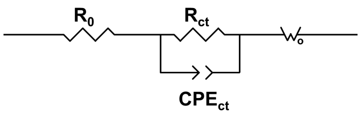	38.42	103.9	/
PEO-$LiClO_4$		46.51	195.2	/
PEO-LiBOB	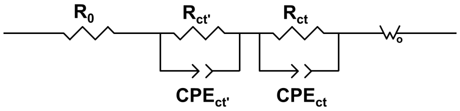	9.117	971.1	111.6

[b)] Compared with SPEs with $TFSI^-$, FSI^-, and ClO_4^- as anions, the ionic cluster aggregation in the PEO-LiBOB lead a distinct multiple charge-transfer interface (the multi semi-circles in the high-frequency and mid-frequency regions of EIS results, Figure 3c). Therefore, different equivalent circuits are used for fitting.

Supplementary Table 4. Redox peaks of CV and the ionic conductivities of different SPEs

SPE of Li||PVF cells	$E_{\text{Oxidation peak}}$	$E_{\text{Reduction peak}}$	$\Delta E_{Ox.p\text{-} Red.p}$	Ionic conductivity at 60 °C ($S\ cm^{-1}$)
PEO-LiTFSI	3.515	3.412	0.103	3.535E-4
PEO-LiBOB	3.571	3.361	0.210	3.018E-4
PEO-LiFSI	3.622	3.324	0.298	2.160E-4
PEO-$LiClO_4$	3.539	3.227	0.312	7.301E-5

Supplementary Table 5. DFT calculations of binding energies of anion and ethyl ferrocenium

ethyl ferrocenium absolute energy (a.u.)	anion species	anion absolute energy (a.u.)	compound absolute energy (a.u.)	$E_{binding}$ (kcal mol^{-1})	E_0 [c)]
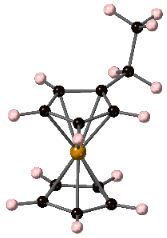	$TFSI^-$	-1827.61	-3557.01	-46.05	3.463
	BOB^-	-779.62	-2509.04	-58.92	3.470
	FSI^-	-1351.90	-3081.34	-69.84	3.476
-1729.32	ClO_4^-	-760.93	-2490.38	-75.37	3.381

[c)] mean of oxidation and reduction peaks (($E_{Ox.p}$+$E_{Red.p}$) /2) represent the electrode potential (E_0)

Supplementary Table 6. Performance comparison between this work and the reported literature

Design of solid polymer electrolytes	Ionic conductivity ($S\ cm^{-1}$) at test temperature	Thickness of solid/gel electrolyte	Cathode / Theoretical capacity ($mAh\ g^{-1}$) [d)]	Amount and mass loading of positive electrode (active materials)	Current Density ($\mu A\ cm^{-2}$) / Capacity Retention	Ref.
Unmodified PEO-LiTFSI	3.5×10^{-4} at 60 °C	100 µm	Polyvinyl ferrocene / 124	40 wt.%, 1.0 mg cm^{-2} for long cycles; (60 wt.%, 9.6 mg for PVF+$LiFePO_4$ mixed high mass loading)	100 / 87.1%<br>1000 / 62.9%	This work
PEO-LiTFSI/PMA-LiTFSI	2.05×10^{-4} at 65 °C	130 µm	$LiCoO_2$ / ~140 (Charge to 4.25 V)	70 wt.%, 5.0 mg cm^{-2}	50 / 90.0%<br>500 / 40.7%	8
Cross-linked UIO-66, PETMP and PEGDA	2.26×10^{-4} at 40 °C	80 µm	$LiFePO_4$ / 170	80 wt.%, 2.4 mg cm^{-2}	40.8 / 89.7%<br>408 / 54.7%	9
Ultrathin PI film filled with PEO-LiTFSI	2.3×10^{-4} at 60 °C	8.6 µm	$LiFePO_4$ / 170	60 wt.%, 1.5 mg cm^{-2}	25.5 / 85.3%<br>255 / 44.1%	10
18 wt.% DMF in xPTHF	1.2×10^{-4} at 70 °C	100 µm	$LiFePO_4$ / 170	70 wt.%, 2.5 mg cm^{-2}	42.5 / 73.5%<br>425 / 15.4%	11
PEO_m-$Li_{21}Si_5$ composite SPE	5.6×10^{-4} at 45 °C	100 µm	$LiFePO_4$ / 170	80 wt.%, 1.5 mg cm^{-2}	12.7 / 96.7%<br>510 / 49.7%	12
Polyeutectic electrolyte constructed by NIPAM	7×10^{-4} at 50 °C	100 µm	$LiFePO_4$ / 170	70 wt.%, 3.5 mg cm^{-2}	119 / 85.3%<br>1190 / 44.1%	13
Single Li-ion Conductive Solid Polymer Electrolyte	~10^{-4} at 70 °C	100 µm	$LiFePO_4$ / 170	63 wt.%, 4.0 mg cm^{-2}	34 / 88.2%<br>136 / 41.2%	14
Deep eutectic solvent based self-healing electrolyte	1.79×10^{-3} at 25 °C	glass fiber membrane s	$LiMn_2O_4$ / 148	80 wt.%, 1.5 mg cm^{-2}	17.8 / 79.1%<br>355.2 / 70.4	15
Self-Healing Janus Interfaces for LAGP	$>10^{-3}$ at 25 °C	300 µm	$LiMn_2O_4$ / 148	80 wt.%, 1.2 mg cm^{-2}	7.1 / 86.2%<br>71 / 55.5%	16

[d)] All the cell test were performed with coin-type cell, and lithium metal as negative electrode.

Supplementary Reference List

1. Smith, C. P., & White, H. S. Theory of the interfacial potential distribution and reversible voltammetric response of electrodes coated with electroactive molecular films. *Anal. Chem.* **64**, 2398-2405 (1992).
2. Nerngchamnong, N., Thompson, D., Cao, L., Yuan, L., Jiang, L., Roemer, M., & Nijhuis, C. A. Nonideal electrochemical behavior of ferrocenyl-alkanethiolate SAMs maps the microenvironment of the redox unit. *J. Phys. Chem. C* **119**, 21978-21991 (2015).
3. Zhang, L., Vogel, Y. B., Noble, B. B., Goncales, V. R., Darwish, N., Brun, A. L., ... & Ciampi, S. TEMPO monolayers on Si (100) electrodes: electrostatic effects by the electrolyte and semiconductor space-charge on the electroactivity of a persistent radical. *J. Am. Chem. Soc.* **138**, 9611-9619 (2016).
4. Vogel, Y. B., Zhang, L., Darwish, N., Gonçales, V. R., Le Brun, A., Gooding, J. J., ... & Ciampi, S. Reproducible flaws unveil electrostatic aspects of semiconductor electrochemistry. *Nat. Commun.* **8**, 1-9 (2017).
5. Sheridan, M. V., Gamm, P., Schneebeli, S. T., Breuer, R., Schmittel, M., & Geiger, W. E. Effect of large electrolyte anions on the sequential oxidations of bis (fulvalene) diiron attached to glassy carbon by an ethynyl linkage. *Langmuir* **34**, 1327-1339 (2018).
6. Lindström, H., Södergren, S., Solbrand, A., Rensmo, H., Hjelm, J., Hagfeldt, A., & Lindquist, S. E. Li+ ion insertion in TiO2 (anatase). 2. Voltammetry on nanoporous films. *J. Phys. Chem. B* **101**, 7717-7722 (1997).
7. Song, Z., Zhan, H., & Zhou, Y. Anthraquinone based polymer as high performance cathode material for rechargeable lithium batteries. *Chemical communications* **4**, 448-450 (2009).
8. Zhou, W., Wang, Z., Pu, Y., Li, Y., Xin, S., Li, X., ... & Goodenough, J. B. Double-layer polymer electrolyte for high-voltage all-solid-state rechargeable batteries. *Adv. Mater.* **31**, 1805574 (2019).
9. Wang, H., Wang, Q., Cao, X., He, Y., Wu, K., Yang, J., ... & Sun, X. Thiol-branched solid polymer electrolyte featuring high strength, toughness, and lithium ionic conductivity for lithium-metal batteries. *Adv. Mater.* **32**, 2001259 (2020).
10. Wan, J., Xie, J., Kong, X., Liu, Z., Liu, K., Shi, F., ... & Cui, Y. Ultrathin, flexible, solid polymer composite electrolyte enabled with aligned nanoporous host for lithium batteries. *Nat. Nanotechnol.* **14**, 705-711 (2019).
11. Mackanic, D. G., Michaels, W., Lee, M., Feng, D., Lopez, J., Qin, J., ... & Bao, Z. Crosslinked poly (tetrahydrofuran) as a loosely coordinating polymer electrolyte. *Adv. Energy Mater.* **8**, 1800703 (2018).
12. Liu, Y., Hu, R., Zhang, D., Liu, J., Liu, F., Cui, J., ... & Zhu, M. Constructing Li-Rich Artificial SEI Layer in Alloy-Polymer Composite Electrolyte to Achieve High Ionic Conductivity for All-Solid-State Lithium Metal Batteries. *Adv. Mater.* **33**, 2004711 (2021).
13. Zhang, C., Niu, Z., Bae, J., Zhang, L., Zhao, Y., & Yu, G. Polyeutectic-based stable and effective electrolytes for high-performance energy storage systems. *Energy & Environ. Sci.* **14**, 931-939 (2021).
14. Martinez-Ibañez, M., Sanchez-Diez, E., Qiao, L., Zhang, Y., Judez, X., Santiago, A., ... & Zhang, H. Unprecedented Improvement of Single Li-Ion Conductive Solid Polymer Electrolyte Through Salt Additive. *Adv. Funct. Mater.* **30**, 2000455 (2020).
15. Jaumaux, P., Liu, Q., Zhou, D., Xu, X., Wang, T., Wang, Y., ... & Wang, G. Deep-Eutectic-solvent-based self-healing polymer electrolyte for safe and long-life lithium-metal batteries. *Angew. Chem. Int. Ed.* **59**, 9134-9142 (2020).
16. Liu, Q., Zhou, D., Shanmukaraj, D., Li, P., Kang, F., Li, B., ... & Wang, G. Self-healing Janus interfaces for high-performance LAGP-based lithium metal batteries. *ACS Energy Lett.* **5**, 1456-1464 (2020).
